# Risk Factors for Mortality in Nursing Home Residents: An Observational Study

**DOI:** 10.3390/geriatrics5040071

**Published:** 2020-10-08

**Authors:** José Fermín García-Gollarte, María Montero García-Andrade, Sebastiá J. Santaeugenia-González, José Carlos Solá Hermida, Susana Baixauli-Alacreu, Francisco José Tarazona Santabalbina

**Affiliations:** 1 Medical Department Grupo Ballesol, Universidad Católica de Valencia, La Eliana, 46183 Valencia, Spain; medico.patacona@ballesol.es; 2 Medical Department Grupo Ballesol, La Cala de La Vila, 03570 Alicante, Spain; maria.montenro@ballesol.es; 3 Chronic Care Program, Ministry of Health, Central Catalonia Chronicity Research Group (C3RG), Centre for Health and Social Care, 08500 Barcelona, Spain; sjsantaeugenia@gmail.com; 4 Department of Nursing, Universidad Católica de Valencia San Vicente Mártir, 46001 Valencia, Spain; susanaba@mail.ucv.es; 5 Geriatric Service, Hospital Universitario de la Ribera, 46600 Alzira, Spain; fjtarazonas@gmail.com; 6 Division of Geriatric Medicine, 7GPR+3M Doha, Qatar; 7 CIBERFES, Centro de Investigación Biomédica en Red Fragilidad y Envejecimiento Saludable, 0 28029 Madrid, Spain

**Keywords:** elderly, mortality, nursing homes, risk factors

## Abstract

**Purpose:** Identifying mortality risk factors in people living in nursing homes could help healthcare professionals to individualize or develop specific plans for predicting future care demands and plan end-of-life care in this population. This study aims to identify mortality risk factors in elderly nursing home (NH) residents, based on variables adapted to this environment, routinely collected and easily accessible to their healthcare professionals. **Methods:** A prospective, longitudinal, observational study of NH residents aged 65 years and older was carried out collecting sociodemographic, functional and cognitive status, nutritional variables, comorbidities, and other health variables. These variables were analyzed as mortality risk factors by Cox proportional hazard models. **Results:** A total of 531 residents (75.3% female; average age 86.7 years (SD: 6.6)) were included: 25.6% had total dependence, 53.4% had moderate to severe cognitive impairment, 84.5% were malnourished or at risk of malnutrition, and 79.9% were polymedicated. Risk of mortality (hazard ratio, HR) increased in totally dependent residents (HR = 1.52; *p* = 0.02) and in those with moderate or severe cognitive impairment ((HR = 1.59; *p* = 0.031) and (HR = 1.93; *p* = 0.002), respectively). Male gender (HR = 1.88; *p* < 0.001), age ≥80 years (HR = 1.73; *p* = 0.034), hypertension (HR = 1.53; *p* = 0.012), atrial fibrillation/arrhythmia (HR = 1.43; *p* = 0.048), and previous record of pneumonia (HR = 1.65; *p* = 0.029) were also found to be mortality drivers. **Conclusion:** Age and male gender (due to the higher prevalence of associated comorbidity in these two variables), certain comorbidities (hypertension, atrial fibrillation/arrhythmia, and pneumonia), higher functional and cognitive impairment, and frequency of medical emergency service care increased the risk of mortality in our study. Given their importance and their easy identification by healthcare professionals in nursing homes, these clinical variables should be used for planning care in institutionalized older adults.

## 1. Introduction

Population ageing is contributing to the increasing number of people living in nursing homes (NHs), where they also receive the necessary care in the final stages of life [1].

In Spain, the characteristics of the population living in NHs include an average age of over 80 years, high comorbidity, high functional dependence, and severe cognitive impairment [2]. These patients have a complex relationship with healthcare services, resulting in a higher rate of emergency service visits and acute care hospital admissions [3], with longer stays and worse progression compared to elderly people living in their own homes. Accurate determination of their prognosis would be of great value in providing appropriate care for this population, ensuring trained staff and efficiently allocating the necessary NH resources [2]. Knowledge of mortality drivers, survival rates and residents’ health status are critical to NH directors [4,5]. In this setting, a lack of recognition of advanced chronic diseases or the proximity of death by healthcare professionals has a significant impact on residents’ lives. This may lead to improper diagnoses and treatments, including inadequate hospitalization and futile treatments, as well as a lack of adaptation of the care provided [6,7]. It is estimated that about three-quarters of deaths in the elderly after a long period of chronic disease can be predicted [8]. Accordingly, healthcare professional interventions could aim to reduce risk factors of early mortality, estimate future care requirements in this population, and to consider, in short, the integration of palliative care in NHs [9].

However, due to the presence of multiple conditioning variables of poor prognosis, such as age, gender, functional disability, the presence of pressure ulcers, malnutrition, and dementia, the evaluation of mortality risk factors in this population is complex [10,11].

Several prognostic indices have been developed to predict the risk of mortality in the elderly population [10,12]. However, these indices have several limitations. Many of them were obtained using large databases, which lack accuracy in some of the reported variables compared to the accuracy provided by reviewing medical records [13]. Besides, they consider information that may not be commonly evaluated in routine clinical practice [14]. Prognostic indices often use complex formulas, which may not be clinically practical [15]. Moreover, they have not been validated or prospectively tested in large population samples. Consequently, these tools for the prediction of mortality have not been sufficiently studied or implemented in clinical practice within the social and healthcare environment [14,16].

Since risk factor studies have traditionally focused mainly on the hospital and community environments [14], or particularly on NH residents with specific diseases [17], little evidence is available in this setting, while the number of elderly people living in NHs continues to grow. Mortality risk factors should be easy and commonly assessed by NH staff to ensure the future needs for this population.

The main objective of this study was to identify potential mortality risk factors in institutionalized elderly adults, based on variables adapted to this environment, routinely collected and easily accessible to their healthcare professionals. These could be used to individualize the benefit of acute hospital care or emergency service transfer in case of acute medical crises and better plan their end-of-life care.

## 2. Methods

### 2.1. Design and Participants

A prospective, longitudinal, multi-center, observational cohort study was carried out in NHs for the elderly in the cities of Valencia and Alicante. The study included a total of eight NHs with similar assistance and organizational structures, catering for a total of 700 residents. In these centers, the main assistance functions are performed following common protocols; healthcare records are updated and stored using the same computer program and each center has the same professional teams and professional rate per patient.

From January 2016 to March 2017, residents over 65 years of age, who were expected to reside permanently for at least 15 months, were included. Elderly adults defined as day-care patients, namely those using just some of the center services for a few hours, such as physiotherapy, patients admitted to the NH for a period of less than one year, and with a life expectancy of less than six months, estimated by the eprognosis calculator (https://eprognosis.ucsf.edu/calculators/#/), were excluded.

### 2.2. Outcome Measures

A 15-month follow-up period was established for participants. Overall survival was defined as the time from the initial assessment to the date of death or the last date of follow-up in the case of censored data.

At the beginning of the study, sociodemographic variables (age and gender) and the following variables were recorded.

#### 2.2.1. Functional Status Indicators

Functionality was assessed using the Barthel index [18], which evaluates the basic activities of daily life and determines the patient’s autonomy to carry out these activities. Based on their score, patients were classified as having total dependence (score <20), severe dependence (20–35), moderate dependence (40–55), mild dependence (60–95), or independence (100).

The risk of pressure ulcers (PUs) was evaluated using the Norton scale [19], with a total score range of 1 to 20 points, where a score of ≥14 points indicates that the patient is at risk of developing PUs.

#### 2.2.2. Cognitive Status Indicators

The mental and emotional assessment was performed using the Pfeiffer Short Portable Mental Status Scale [20], considering cognitive impairment to be present when over three errors were recorded. Participants were classified as: normal cognitive state (<3 errors), mild impairment (3–4 errors), moderate impairment (5–7 errors), and severe impairment (≥8 errors). The Pfeiffer scale is a simple and fast screening scale widely used by centers taking part in this study. The test has a very good sensitivity (85.7%), good specificity (79.3%) [20], and a very good correlation with other cognitive performance tests, such as the Cognoscitive mini-test 0.74 [21] (very similar to the Folstein Mini Mental State Examination (MMSE) test).

#### 2.2.3. Nutritional Assessment

The nutritional status was evaluated using the Global Leadership Initiative on Malnutrition (GLIM) criteria [22], and the Mini Nutritional Assessment (MNA) [23]. Patients were considered to be at nutritional risk if their scores were between 17 and 23.5, while malnutrition corresponded to scores below 17.

To determine whether there was weight loss, residents’ weights were recorded at the beginning and at 12 months. A reduction of ≥5 kg without significant changes in the diet was established as weight loss.

Dysphagia was evaluated using the bolus volume–viscosity test [24]. Dysphagia was considered not present if there were no signs of alteration in the safety and efficacy of swallowing.

#### 2.2.4. Comorbidities

In order to determine the presence of comorbidities, the Charlson index adjusted by age [25] was applied to data from the residents’ medical records. A score between 0 and 1 indicated a lack of comorbidity, 2 points indicated low comorbidity, and scores equal to or greater than 3 points indicated high comorbidity.

Residents with values of HbA1c greater than 7.0–7.5% were considered to have diabetes mellitus type II, typical of frail people and with high risk of mortality [26].

Participants were recorded as having high blood pressure (HBP) if their systolic blood pressure was higher than 140 mm of Hg and diastolic blood pressure higher than 90 mm of Hg, measured three times with an interval of five minutes between readings over at least two different visits [27].

The following pathologies were recorded, in the form of dichotomous variables: atrial fibrillation (AFib)/arrhythmias, kidney failure, Parkinson’s disease, and cancer. In addition, previous events that could affect the general condition of the patient were recorded, such as pneumonia, anaemia, urinary infection, and hip fractures in the last two years.

#### 2.2.5. Other Health Variables

Physical performance was assessed by the timed up and go (TUG) test, to correctly identify people with and without risk of suffering a fall. This is a simple test in which the resident starts in a sitting position, gets up, walks 3 m, turns around and goes back to the chair to sit down again. Taking more than 20 s to perform this test indicates a high risk of suffering a fall [28].

In addition, a record was made of the number of falls, use of emergency services, hospitalizations, and episodes of delirium suffered by residents during the last year detected using the confusion assessment method (CAM) [29] in each shift. Information as to the type of incontinence presented by participants (urinary, fecal, or mixed) was also included.

The Visual Analogue Scale (VAS) [30] was used to assess pain in patients with preserved cognitive skills. The Pain Assessment in Advanced Dementia (PAINAD) [31] scale was used for those with cognitive impairment and inability to respond. Poor pain control is indicated by scores above 3 when using the VAS scale and by scores above 4 when using the PAINAD scale.

Finally, frailty was assessed using the Edmonton Frailty Scale, which evaluates nine domains (cognition, general health status, functional independence, social support, medications, nutrition, mood, continence, functional performance). Participants with scores between 0 and 5 were considered to be non-frail; 6–7, vulnerable; 8–9, mildly frail; 10–11, moderately frail; and 12–17, severely frail [32].

### 2.3. Pharmacological Treatment

Polypharmacy was recorded when participants took ≥5 medications for chronic conditions [33]. The frequencies of the use of anticholinergics, anxiolytics, antidepressants, and neuroleptics were also recorded.

### 2.4. Data Analysis

Absolute and relative frequencies were calculated to describe the distribution of categorical variables. The mean and standard deviation (SD) or median and interquartile range (IQR) were calculated, according to data distribution for the quantitative variables.

The Kaplan–Meier method was used to estimate overall survival at 12 months. Survival functions were estimated according to functional status and cognitive impairment and the logrank test was used to compare survival functions.

Our outcome variable was time to death, so in order to analyze mortality risk factors, hazard ratios and the 95% confidence interval were estimated using univariate and multivariate Cox proportional hazard models. In univariate analysis, all possible factors were explored. The variables to be included in the multivariate model were selected, taking into account the following criteria: a statistical significance of *p* < 0.1 in univariate analysis; the absence of collinearity; and the clinical evaluation of the physician.

Starting from the model that included all the above variables, models were adjusted successively and variables were discarded manually, until the simplest possible model was obtained with the smallest number of variables (parsimony principle) and highest predictive capacity. The predictive capacity of the multivariate model obtained was estimated using Harrell’s C-index (concordance statistic), a C-index of 0.5 is equivalent to random prediction whereas an index of 1 corresponds to a perfect prediction.

All tests were calculated bilaterally, and the threshold of significance was established at a value of *p* < 0.05.

The data were analyzed using the statistical package SPSS version 17 and STATA version 12 (SAS Institute, Inc., Cary, NC, USA).

### 2.5. Ethical Considerations

The study was evaluated and approved by the Ethics Committee of the Universidad Católica de Valencia “San Vicente Mártir” and led by the medical staff of participating NHs. It was carried out with the collaboration of an external group of researchers trained in the anonymous periodic collection of participant data using coded lists preventing researchers from discovering the actual identity of patients. This is in accordance with the data protection regulations [34], and with the principles established in the Declaration of Helsinki [35]. All patients were asked for their written consent to the recording and use of research data upon admission to the nursing home. In the case of patients with advanced dementia, their relatives or caregivers were asked to sign the written consent.

## 3. Results

The study included a total of 531 residents, mainly women (*n* = 400; 75.3%). Their average age was 86.7 years (SD 6.6 years). The results of the main variables at the start of the follow-up are presented in Table 1.

During the 15-month follow-up, 166 residents (31.3%) died. The characteristics of deceased and non-deceased patients are shown in Table 2. The distribution of inter-group variables provides a first indication of the patient profile with the highest mortality risk: male patients with strong dependence and severe cognitive impairment, with a higher risk of PUs, higher Charlson index, and greater Edmonton Frailty Scale score.

Overall survival at 6, 12, and 15 months was determined as 91.0%, 76.8%, and 70.0%, respectively. This mortality rate is similar to that of another study carried out in Catalonia (northeast of Spain) with a large number of centers and cases involved [3].

### Mortality Risk Factors

**Figure 1:**
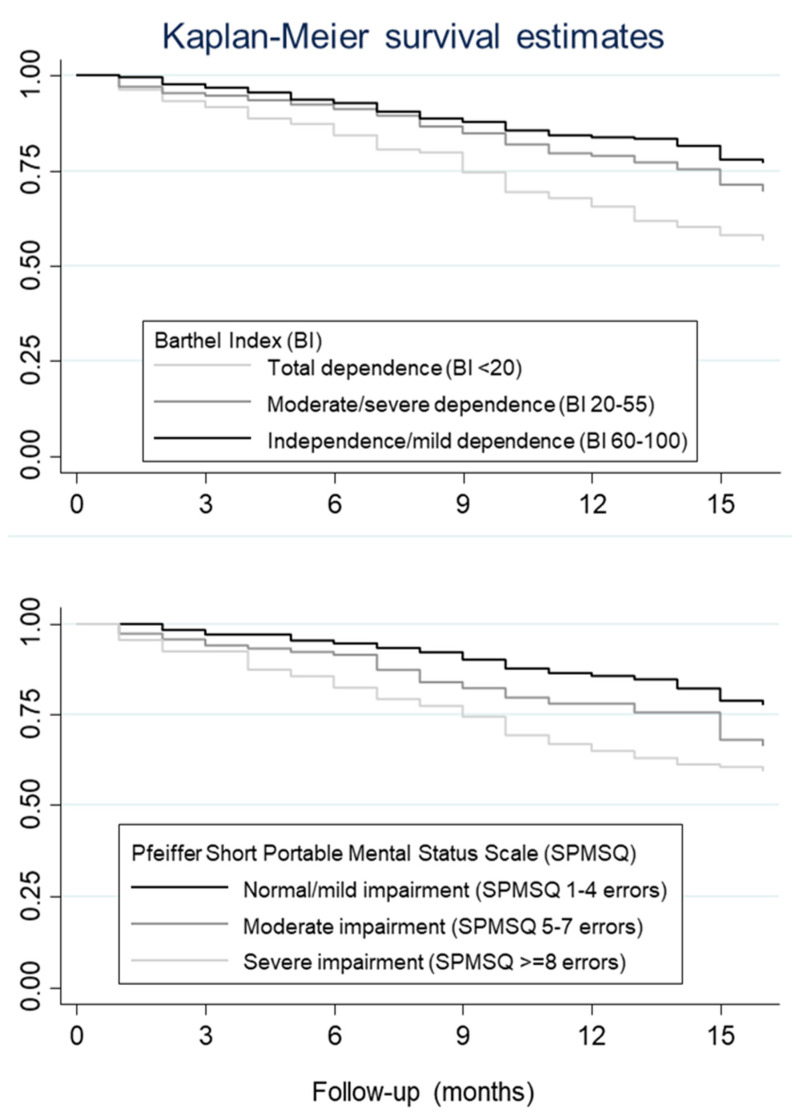
Estimated survival of residents under three scenarios of cognitive impairment and functional dependence.

The 12-month survival was 67.0% in patients with severe cognitive impairment, 80.0% in patients with moderate impairment, and 86.0% in patients with mild impairment. Depending on the degree of dependency, the 12-month survival was 67.0%, 80.0%, and 85.0% in patients with total dependence, moderate/severe, and mild/independent, respectively. Thus, the risk of mortality is significantly raised with the presence of increased cognitive impairment (logrank test <0.001) and increased dependence (logrank test <0.001) (Figure 1).

The hazard ratio (HR) estimated in the univariate Cox model (Table 3), shows that the mortality risk increased with statistical significance in men, as well as in residents with total dependence and moderate and severe cognitive impairment. Patients with dysphagia had a higher mortality risk, but no statistically significant association was found with the degree of malnutrition or weight loss. For comorbidities, HBP and AFib/arrhythmia were significantly associated with increased mortality risk. Patients who had pneumonia, urinary infection, or incontinence also had an increased mortality risk. No association between mortality and pharmacological treatment was observed, except in residents with antidepressant treatment, where a lower risk of death was observed. The number of times emergency services were used over the past year only correlated with mortality when the number was ≥ 2.

The factors identified in the univariate Cox model were included in the multivariate analysis. The multivariate model of mortality predictors is presented in Table 4. Gender, age, degree of dependence, cognitive impairment, HBP, AFib/arrhythmia, and pneumonia were identified as independent risk factors for mortality. Thus, male gender and severe cognitive impairment almost doubled the risk of death, with HRs of 1.88 and 1.93, respectively. Harrel’s C-index for this model was 0.67, indicating a moderate discriminatory capacity.

## 4. Discussion

This study provides useful information on the prognostic factors of mortality in the elderly institutionalized population from variables routinely collected in clinical practice, which may serve to improve the clinical management and individual planning of these patients’ care.

In our study, the univariate Cox model showed that frequent complications in elderly patients such as dysphagia or pneumonia significantly increased the mortality risk of institutionalized individuals. These results are consistent with those obtained in an observational study carried out in Spain in NHs, where pneumonia was identified as one of the most serious diseases and as a predictor of mortality in institutionalized patients with dysphagia [36]. Accurate and systematic diagnosis of these diseases would help improve resident management and Quality of Life (QoL) by facilitating a timely and appropriate approach to these complications.

Other geriatric syndromes such as incontinence and urinary infection also showed predictive mortality capacity in the univariate model. Although these indicators present a lower risk of mortality than pneumonia, their predictive capacity for mortality is in line with previous research showing a correlation between the incidence of urinary incontinence in residents and mortality [37]. In this research, elderly people suffering from severe incontinence presented a high risk of mortality. Another study showed that urinary infections were the most common cause of bacteremia acquired in NHs [38], therefore addressing urinary infections is relevant for resident care.

Our results show that mortality risk increased significantly in line with greater dependency. These results are consistent with those carried out in the hospital environment, which indicate the need for assistance in carrying out daily activities as a predictive factor of mortality [39].

The risk of mortality also increased in residents with more severe cognitive impairment. Although severe cognitive impairment has been linked to complications associated with a terminal illness, such as dysphagia, febrile episodes, or pneumonia [36,40], previous studies indicate that these complications are important mortality risk factors in all stages of dementia; thus, in mortality prediction, we should analyze not only the severity of dementia [40] but also the presence of these complications in all their phases.

According to the multivariate Cox model, in addition to gender, age, degree of cognitive impairment, and level of dependency, other factors of special importance were cardiovascular comorbidities, such as HBP, AFib, and arrhythmia. These chronic conditions are often associated with dementia in the elderly population and are risk factors for other serious clinical conditions, such as congestive heart failure, which also reduces survival. These chronic conditions have been previously associated with mortality in both newly admitted and long-term patients in NHs [41]. On the other hand, the elderly population with multi-morbidity requires the concomitant use of multiple medications. Previous studies show that in this population, polymedication is associated with an increase in drug interactions and adverse events [42,43]. Thus, medications with sedative or anticholinergic properties cause an increased risk of cognitive and functional deterioration, greater deterioration in the activities of daily life, an increase in hospital admissions, and mortality [44,45]

However, in our study, the univariate model did not show a statistically significant association between polymedication and mortality. This absence of a significant association between these variables may be because approximately 80% of the subjects were polymedicated or that the prescribed drugs were adequate in a context of high comorbidity.

A recent study has identified the relationship between demographic and medication-related predictors of mortality in older populations. It shows that a combination of demographics, chronic disease, and medications predicted mortality [46].

Concerning the discriminatory capacity of the model, a C-index of about 0.7 was obtained, similar to other mortality predictive models of elderly patients in NHs [14], and it suggests unexplained variation in mortality. The discriminatory ability of our index, like others previously obtained in nursing homes is, however, consistent with other indices that commonly drive clinical decisions, such as: the CHADS2 index to help determine warfarin therapy (C-index 0.68–0.72); the Framingham risk score to help determine lipid therapy (C-index 0.63–0.83); and the TIMI risk score to help determine invasive therapy for unstable angina (C-index 0.65). Compared to other published predictive models, our results presented a lower C-index but included only eight variables (sex, age, high blood pressure, atrial fibrillation, moderate and severe cognitive impairment, severe functional impairment, previous pneumonia diagnosis, emergency department visits).

For example, the first version of the Minimum Data Set Mortality Risk Index (MMRI) got a better C-index (0.75) but included fourteen variables [47]. The simplified MMRI used in our study included eight variables and had a better score (receiver operating characteristic curve (ROC) area 0.76) when predicting six-month mortality. Likewise, MDS-CHESS 3.0 included twelve variables and its C-index was lower (0.655) than the one obtained in our study [14]. Finally, Minimum Data Set 3.0 (MDS 3.0) improved prediction for 30- and 60-day mortality (C-index of 0.744 and 0.709, respectively) but required more variables (seventeen) [48].

Despite having a predictive capacity similar to other scales in use in routine clinical practice, we consider that a C-index of 0.67 is a low value to develop a predictive score from our multivariate model. A C-index of at least 0.8 would be desirable.

Our results suggest that by using the data usually recorded in clinical histories, nursing home staff could identify higher mortality risk in residents. This information could be very useful in establishing palliative care programs and advanced care plans.

Nevertheless, our study has some important limitations that must be reported. First of all, there was some bias due to the total missing values rate of 15%. It is also important to point out that one limitation of the study is the imbalance between the number of women and men included in the sample. The proportion of men was probably lower due to their lower survival rate compared to women; furthermore, given the high degree of dependence of the study population, it should also be noted that women survive longer than men when faced with severe disability [49]. Likewise, the authors are aware of the fact that the TUG test score, as a simple numerical result, is only partially valid as a detection method for falls and it was not possible to analyze how the gait was disturbed or if it was possible to correct this disturbance.

## 5. Conclusions

This study shows that, at the end of life, serious functional and cognitive impairments are of great importance, relating directly to increased mortality. Prognostic factors identified using variables that are easily accessible to healthcare professionals and adapted to this environment provide information of great relevance for planning care and improving resident QoL during their end-of-life phase.

## Figures and Tables

**Table 1:** Description of the sample according to the number of residents (percentage).

**Sociodemographic and Functional Variables**										***n*** **(%)**					
**Gender**		Female								400 (75.3%)					
		Male								131 (24.7%)					
**Age**		<80 years								86 (16.2%)					
		≥80 years								445 (83.8%)					
**Origin of residents**		Home								313 (59%)					
		Hospital discharge								160 (30%)					
		Other nursing home								58 (11%)					
**Reason for admission**		Increased intensity of care								414(78%)					
		Post-surgical care								61 (12%)					
		Other reasons								56 (10%)					
**Length of stay (months)**		Mean (SD)								35.46 (61.83)					
**Dependency level (Barthel index)**		Total (<20)		Severe (20–35)			Moderate (40–55)				Mild (60–95)				Independent (100)
		135 (25.6%)		87 (16.5%)			84 (15.9%)				203 (38.4%)				19 (3.6%)
**Risk of pressure ulcers (Norton)**		Very high (5–9)			High (10–12)				Medium (13–14)					Minimum/no risk (>14)	
		17 (3.2%)			69 (13.1%)				110 (20.8%)					332 (62.9%)	
**Cognitive Status Indicators**															
**Pfeiffer Short Portable Mental Status Scale**		Normal (<3 errors)			Mild impairment (3–4 errors)				Moderate impairment (5–7 errors)					Severe impairment (≥8 errors)	
		169 (32.3%)			75 (14.3%)				119 (22.7%)					161 (30.7%)	
**Assessment of Nutritional Status**															
**BMI**		Mean (SD)								26.64 (5.45)					
**MNA**		Malnourished (<17)				At risk (17–23)						Well nourished (≥24)			
		177 (33.6%)				268 (50.9%)						82 (15.6%)			
**Weight loss (>5 kg in 12 months)**		88 (18.5%)													
**Incidence of dysphagia**		115 (21.8%)													
**Comorbidities**															
**Charlson**		Median (IQR)								7 (6–9)					
		Charlson ≥ 5								289 (94.1%)					
**AFib/Arrhythmia**		141 (26.7%)													
**Chronic heart failure**		217(40.9%)													
**High blood pressure**		314 (59.5%)													
**Kidney failure** **(** **CKD Stage 3 or higher)**		72 (13.7%)													
**Pneumonia**		55 (10.4%)													
**Anemia**		182 (34.5%)													
**Urinary tract infection**		180 (34.2%)													
**Risk Factors and Health Variables**															
**Risk of suffering a fall (TUG > 20 s)**		Normal								223 (68.4%)					
		High								103 (31.6%)					
**Hip fracture**		93 (17.6%)													
**Falls/last year**		1								94 (17.8%)					
		≥2								74 (14%)					
**Emergency service/last year**		1								82 (15.7%)					
		≥2								29 (5.6%)					
**Hospital./last year**		1								89 (17.1%)					
		≥2								36 (6.9%)					
**Ep. delirium/last year**		1								34 (8.9%)					
		≥2								30 (7.9%)					
**Incontinence**		Urinary incontinence				Fecal incontinence						Mixed incontinence			
		147 (28.1%)				19 (3.6%)						196 (37.5%)			
**Pain (VAS or PAINAD ≥4)**		146 (27.5%)													
**Edmonton Frailty Scale**		Median (IQR)								9 (6–13)					
**Pharmacological treatment**															
**Polymedication (≥5 treatment)**	Anticholinergics		Anxiolytics					Anti-depressant					Neuroleptics		
**422 (79.9%)**	98 (19.2%)		191 (37.3%)					162 (31.6%)					115 (22.4%)		

Categorical variables are expressed as count and percentages, and quantitative variables are characterized in terms of mean and standard deviation or median and interquartile range. BMI, body mass index; MNA, Mini Nutritional Assessment; IQR, interquartile range; AFib, atrial fibrillation; CKD, chronic kidney disease; TUG, timed up and go test; Hospital., hospitalizations; Ep. delirium, episodes of delirium detected using the confusion assessment method; VAS, Visual Analogue Scale; PAINAD, Pain Assessment in Advanced Dementia.

**Table 2:** Description of the sample according to its distribution in deceased and non-deceased.

			Alive *n* (%)	Expired *n* (%)
			*n* = 365	*n* = 166
**Sociodemographic and functional variables**	Gender	Female	286 (78.3%)	114 (68.6%)
		Male	79 (21.6%)	52 (31.3%)
	Age	<80 years	67 (18.3%)	19 (11.4%)
		≥80 years	298 (81.6%)	147 (88.5%)
	Dependency level (Barthel index)	Total (<20)	75 (20.5%)	60 (36.8%)
		Severe (20–35)	57 (15.6%)	30 (18.0%)
		Moderate (40–55)	62 (16.9%)	22 (13.5%)
		Mild (60–95)	153 (41.9%)	50 (30.6%)
		Independence (100)	18 (4.9%)	1 (0.6%)
	Risk of pressure ulcers (Norton)	Very high (5–9)	7 (1.9%)	10 (6.1%)
		High (10–12)	34 (9.3%)	35 (21.4%)
		Medium (13–14)	70 (19.1%)	40 (24.5%)
		Minimum/no risk (>14)	254 (69.5%)	78 (47.8%)
**Cognitive status indicators**	Pfeiffer Short Portable Mental Status Scale	Normal (<3 errors)	132 (36.3%)	37 (22.9%)
		Mild impairment (3–4 errors)	57 (15.7%)	18 (11.2%)
		Moderate impairment (5–7 errors)	79 (21.7%)	40 (24.8%)
		Severe impairment (≥8 errors)	95 (26.1%)	66 (40.9%)
**Assessment of nutritional status**	BMI	Mean (SD)	27.19 (29.78)	25.41 (27.97)
	MNA	Malnourished (< 17)	133 (36.5%)	44 (26.9%)
		At risk (17–23)	174 (47.8%)	94 (57.6%)
		Well nourished (≥24)	57 (15.6%)	25 (15.3%)
	Weight loss (>5 kg in 12 months)		53 (16.3%)	35 (23.1%)
	Incidence of dysphagia		66 (18.0%)	49 (30.0%)
**Comorbidities**	Charlson	Median (IQR)	7 (6–8)	8 (6–9)
	AFib/Arrhythmia		71 (19.5%)	46 (28.2%)
	Chronic heart failure		141 (38.8%)	76 (45.2%)
	High blood pressure		209 (57.2%)	105 (64.4%)
	Kidney failure (CKD stage 3 or higher)		45 (12.3%)	27 (16.6%)
	Diabetes mellitus		83 (22.7%)	50 (30.7%)
	Parkinson’s		26 (7.2%)	15 (9.4%)
	Cancer		44 (12.1%)	27 (16.6%)
	Pneumonia		31 (8.4%)	24 (14.7%)
	Anemia		117 (32.0%)	65 (39.8%)
	Urinary tract infection		113 (31.0%)	67 (41.1%)
**Risk factors and health variables**	Risk of suffering a fall (TUG > 20 s) (*n* = 326)	High	78 (31.84%)	25 (30.86%)
	Hip fracture		67 (18.3%)	26 (15.9%)
	Falls/last year	1	68 (18.6%)	26 (16.0%)
		≥2	48 (13.1%)	26 (16.0%)
	Emergency service/last year	1	55 (15.3%)	27 (16.5%)
		≥2	16 (4.4%)	13 (7.9%)
	Hospital./last year	1	58 (16.2%)	31 (19.0%)
		≥2	22 (6.1%)	14 (8.5%)
	Ep. delirium/last year	1	27 (10.4%)	7 (5.7%)
		≥2	19 (7.3%)	11 (9.0%)
	Incontinence	Urinary incontinence	103 (28.4%)	44 (27.3%)
		Fecal incontinence	12 (3.3%)	7 (4.3%)
		Mixed incontinence	121 (33.4%)	75 (46.5%)
	Pain (VAS or PAINAD ≥ 4)		102 (28.1%)	44 (26.2%)
	Edmonton Frailty Scale (*n* = 413)	Median (IQR)	9 (6–12)	10 (7–14)
		Not frail (0–5)	70 (24.73%)	17 (14.78%)
		At risk (6–7)	31 (10.95%)	23 (20%)
		Pre-frail (8–9)	48 (16.96%)	19 (16.52%)
		Moderately frail (10–11)	53 (18.73%)	16 (13.91%)
		Frail (12–17)	81 (28.62%)	40 (34.78%)
**Pharmacological treatment**	Polymedication (≥5 treatment)		287 (78.6%)	135 (82.8%)
	Anticholinergics		70 (19.8%)	28 (17.8%)
	Anxiolytics		129 (36.5%)	62 (38.9%)
	Anti-depressant		122 (34.4%)	40 (25.1%)
	Neuroleptics		79 (22.3%)	36 (22.6%)

Categorical variables are expressed as count and percentages, and quantitative variables are characterized in terms of mean and standard deviation or median and interquartile range. SD, standard deviation; BMI, body mass index; MNA, Mini Nutritional Assessment; AFib, atrial fibrillation; CKD, chronic kidney disease; TUG, timed up and go test; Hospital., hospitalizations; Ep. delirium, episodes of delirium detected using the confusion assessment method; VAS, Visual Analogue Scale; PAINAD, Pain Assessment in Advanced Dementia; IQR, interquartile range.

**Table 3:** Mortality predictors according to the univariate model.

Risk Factor		HR	CI95% HR		*p*-Value
**Sociodemographic and functional variables**	**Male**	1.45	1.04	2.01	0.027 *
	**≥80 years**	1.53	0.95	2.48	0.079
	**Total dependence (Barthel <20)**	1.93	1.41	2.62	<0.001 *
	**High/very high risk of pressure ulcers (Norton ≤ 12)**	2.51	1.78	3.54	<0.001 *
**Cognitive status indicators**	**Moderate cognitive impairment (SPMSQ 5-7 errors)**	1.58	1.05	2.37	0.028 *
	**Severe cognitive impairment (SPMSQ ≥8 errors)**	2.14	1.50	3.07	<0.001 *
**Assessment of nutritional status**	**BMI**	0.95	0.91	0.99	0.023 *
	**Malnourished (MNA < 17)**	0.76	0.47	1.25	0.282
	**At risk of malnutrition (MNA 17-23)**	1.15	0.74	1.78	0.547
	**Weight loss (> 5 kg in 12 months)**	1.40	0.96	2.04	0.084
	**Dysphagia**	1.74	1.24	2.43	0.001 *
**Comorbidities**	**Charlson ≥7**	1.55	0.98	2.44	0.060
	**AFib/Arrhythmia**	1.56	1.11	2.20	0.010 *
	**Chronic heart failure**	1.28	0.94	1.73	0.114
	**High blood pressure**	1.35	0.98	1.86	0.065
	**Kidney failure (CKD stage 3 or higher)**	1.37	0.91	2.07	0.136
	**Diabetes mellitus**	1.39	0.99	1.94	0.054
	**Parkinson’s**	1.32	0.78	2.25	0.303
	**Cancer**	1.35	0.89	2.04	0.153
	**Pneumonia**	1.62	1.05	2.50	0.029 *
	**Anemia**	1.29	0.94	1.76	0.112
	**Urinary tract infection**	1.44	1.05	1.97	0.022 *
**Risk factors and health variables**	**High risk of falling (TUG > 20 s)**	0.95	0.6	1.53	0.846
	**Hip fracture**	0.86	0.56	1.3	0.472
	**≥2 Falls/last year**	1.16	0.76	1.78	0.491
	**≥2 Hospital./last year**	1.36	0.78	2.37	0.277
	**≥2 Emergency service/last year**	1.67	0.94	2.95	0.080
	**≥2 Ep. delirium/last year**	1.26	0.68	2.35	0.461
	**Urinary or fecal incontinence**	1.72	1.18	2.50	0.004 *
	**Pain (VAS or PAINAD ≥4)**	0.91	0.65	1.29	0.607
	**Edmonton Frailty Scale**	1.05	1.01	1.09	0.018 *
	**At-risk/pre-frail (Edmonton Frailty Scale 6-9)**	1.92	1.09	3.37	0.023 *
	**Moderately frail/frail (Edmonton Frailty Scale 10-17)**	1.61	0.94	2.77	0.085
**Pharmacological treatment**	**Polymedication (≥5 treatment)**	1.26	0.84	1.9	0.263
	**Anticholinergics**	0.88	0.58	1.32	0.535
	**Anxiolytics**	1.08	0.79	1.49	0.621
	**Neuroleptics**	0.99	0.69	1.44	0.975
	**Anti-depressants**	0.69	0.49	0.99	0.046 *

* p < 0.05. Hazard ratio and 95% confidence interval estimated with univariate Cox regression models. HR, hazard ratio; CI95%, 95% confidence interval; SPMSQ, Pfeiffer Short Portable Mental Status Scale; BMI, body mass index; MNA, Mini Nutritional Assessment; AFib, atrial fibrillation; CKD, chronic kidney disease; TUG, timed up and go test; Hospital., hospitalizations; Ep. delirium, episodes of delirium detected using the confusion assessment method; VAS, Visual Analogue Scale; PAINAD, Pain Assessment in Advanced Dementia.

**Table 4:** Mortality predictors according to the multivariate Cox regression model.

Predictor	HR	CI95% HR		*p*-Value
**Male**	1.88	1.32	2.67	<0.001 *
**≥80 years**	1.73	1.04	2.86	0.034 *
**Moderate cognitive impairment (SPMSQ 5-7 errors)**	1.59	1.04	2.43	0.031 *
**Severe cognitive impairment (SPMSQ ≥8 errors)**	1.93	1.28	2.92	0.002 *
**Total dependence (Barthel <20)**	1.52	1.07	2.17	0.02 *
**High blood pressure**	1.53	1.10	2.14	0.012 *
**AFib/Arrhythmia**	1.43	1.00	2.04	0.048 *
**Pneumonia**	1.65	1.05	2.58	0.029 *
**≥2 Emergency service/last year**	1.80	0.96	3.36	0.067

C Index 0.67; * *p* < 0.05. Hazard ratio and 95% confidence interval estimated with multivariate Cox regression models. HR, hazard ratio; CI95%, 95% confidence interval; SPMSQ, Pfeiffer Short Portable Mental Status Scale; AFib, atrial fibrillation.
